# Synovial Sarcoma Microvesicles Harbor the SYT-SSX Fusion Gene Transcript: Comparison of Different Methods of Detection and Implications in Biomarker Research

**DOI:** 10.1155/2016/6146047

**Published:** 2016-03-16

**Authors:** A. Fricke, P. V. Ullrich, A. F. V. Cimniak, M. Follo, S. Nestel, B. Heimrich, I. Nazarenko, G. B. Stark, H. Bannasch, D. Braig, S. U. Eisenhardt

**Affiliations:** ^1^Clinic for Plastic and Hand Surgery, Medical Center - Faculty of Medicine, University of Freiburg, Freiburg, Germany; ^2^Clinic for Hematology, Oncology and Stem Cell Transplantation, Medical Center - Faculty of Medicine, University of Freiburg, Freiburg, Germany; ^3^Institute of Anatomy and Cell Biology, University of Freiburg, Freiburg, Germany; ^4^Institute for Environmental Health Sciences and Hospital Infection Control, Medical Center - Faculty of Medicine, University of Freiburg, Freiburg, Germany

## Abstract

*Background*. Synovial sarcoma is an aggressive soft-tissue malignancy. This study examines the presence of the SYT-SSX fusion transcript in synovial sarcoma microvesicles as well as its potential role as a biomarker for synovial sarcoma.* Patients and Methods*. Microvesicle release of synovial sarcoma cells was examined by transmission electron microscopy. RNA-content was analyzed by qPCR, nested PCR, nested qPCR, and droplet digital PCR to compare their sensitivity for detection of the SYT-SSX fusion gene transcript. Whole blood RNA, RNA of mononuclear cells, and microvesicle RNA of synovial sarcoma patients were analyzed for the presence of the fusion gene transcripts.* Results*. Electron microscopic analysis revealed synovial sarcoma cells releasing membrane-enclosed microvesicles.* In vitro*, the SYT-SSX fusion gene transcript was detected in both synovial sarcoma cells and microvesicles. Nested qPCR proved to be the most sensitive in detecting the SYT-SSX fusion gene mRNA. In contrast, the fusion gene transcript was not detected in peripheral blood cells and microvesicles of synovial sarcoma patients.* Conclusion*. Synovial sarcoma cells release microvesicles harboring the SYT-SSX fusion transcript. Nested qPCR proved to be the most sensitive in detecting the SYT-SSX fusion gene mRNA; however, more sensitive assays are needed to detect cancer-specific microvesicles in the peripheral blood of cancer patients.

## 1. Introduction

Synovial sarcoma is an aggressive soft-tissue malignancy and constitutes one of the largest subgroups of soft-tissue sarcoma, especially in adolescents and young adults [1, 2]. The cytogenetically defined translocation t(X;18)(p11.2;q11.2) found in human synovial sarcoma results in the fusion of the SYT gene on chromosome 18 to SSX1, SSX2, or SSX4 on chromosome X at Xp11.2, leading to the formation of SYT-SSX fusion transcript [3–5]. This fusion transcript competes for assembly with wild-type SS18, forming an altered complex lacking the tumor suppressor BAF47 (hSNF5), resulting in Sox2 activation and leading to proliferation of synovial sarcoma tumor cells [6]. Also, SYT-SSX has been shown to affect polycomb-mediated gene repression and SWI/SNF chromatin remodeling as well as deregulating WNT-*β*-catenin signaling in synovial sarcoma [7]. Interestingly, while expression of the SYT-SSX2 oncoprotein leads to induction of synovial sarcoma with 100% penetrance in immature myoblasts, its expression in more differentiated cells induces myopathy without tumor induction in a mouse model [8].

The presence of the SYT-SSX fusion transcript enables specific and sensitive molecular diagnosis of synovial sarcoma, being detectable in almost all synovial sarcoma tissues [9, 10]. As shown in a recent meta-analysis of 10 studies comprising 902 patients with synovial sarcoma, there are no significant differences in overall survival and disease-specific survival rates between patients with synovial sarcoma expressing the SYT-SSX1 and SYT-SSX2 fusion gene; however, SYT-SSX1 seems to represent an unfavorable prognostic factor of progression- and metastasis-free survival [11]. Nevertheless, the prognostic value of the fusion gene variant for survival still remains a matter of debate [10, 12].

Since it has been shown that tumor cells release small vesicles containing cell-specific proteins, surface markers, and even mRNA variants specific for certain neoplasms such as the EGFRvIII splice variant in glioblastoma [13], the aim of this study is to evaluate whether microvesicles shed by synovial sarcoma cells carry the tumor-specific fusion gene SYT-SSX transcripts. Moreover, this study analyzes the sensitivity of different methods for the detection of the SYT-SSX fusion genes as a potential biomarker for synovial sarcoma.

## 2. Materials and Methods

### 2.1. Cell Culture

Human synovial sarcoma cells (cell line 1273/99, kindly provided by Dr. Marcus Renner, Institute of Pathology, University of Heidelberg), which harbor the SYT-SSX2 fusion gene [14], were cultured in F-12 nutrient mixture (Ham) (Life Technologies, Carlsbad, CA, USA) supplemented with 10% FBS superior and 100 U/mL Penicillin-Streptomycin. THP-1 cells (human acute monocytic leukemia cell line) were cultured in RPMI medium 1640 (with L-Glutamine; Life Technologies) supplemented with 10% FBS superior and 100 U/mL Penicillin-Streptomycin.

### 2.2. Microvesicle-Purification of Synovial Sarcoma Cells

Since fetal bovine serum was described to contain extracellular vesicles which might alter the results of the analysis of tumor microvesicles [15], synovial sarcoma cells were cultured in microvesicle-free medium (DMEM containing 5% microvesicle-depleted fetal bovine serum/dFBS) until 80% confluency, prepared by ultracentrifugation at 110,000 g for 16 h to remove bovine microvesicles as described by Skog et al. [13] and conditioned medium was collected after 48 h. Microvesicles were purified from the conditioned supernatant by differential centrifugation steps as previously described [16]. Briefly, conditioned medium was centrifuged for 10 min at 300 g to eliminate cell contamination. Supernatants were further centrifuged for 20 min at 16,500 g. Microvesicles were then sterile-filtered (0.22 *μ*m) and pelleted by ultracentrifugation at 110,000 g for 80 min. The microvesicle pellets were then washed in PBS, pelleted again at 110,000 g for 80 min, dissolved in PBS, and stored at −80°C until further use.

### 2.3. Transmission Electron Microscopy (TEM)

Synovial sarcoma cells were plated on glass slides in 24-well plates. At 80% confluency, the medium was removed and cells were rinsed and incubated in PBS at 37°C for 90 min in order to trigger microvesicle release. Cells were subsequently washed with PBS and fixed with 0.1 M phosphate buffer (PB) containing 4% paraformaldehyde (PFA) and 2% glutaraldehyde (GA) for 30 min. The cells were then rinsed in ddH_2_O and 0.1 M PB for each 10 min and osmicated (1% OsO_4_ and 6.86% sucrose in 0.1 PB) for 40 min. Thereafter, the cells were rinsed several times in 0.1 M PB, immersed in 50% ethanol (EtOH) for 10 min, incubated in 1% uranyl acetate in 70% EtOH for 35 min, and dehydrated in a graded series of ethanol (90%, 95%, and 100%). After rinsing twice in propylene oxide for 10 min, the glass coverslips with the cells attached on top were immersed in a 1 : 1 mixture of propylene oxide and Durcopan (Fluka, Buchs, Switzerland) for 1 h. This was followed by embedding the cells in Durcopan overnight; then, they were mounted under coverslips with fresh Durcopan and left to incubate for 24 h at 56°C for polymerization. Ultrathin sections (40 nm) were cut, collected on Formvar-coated nickel grids, and digitally photographed (LEO 906 E, Zeiss, Oberkochen, Germany) using SIS software (Olympus, Hamburg, Germany). The method was previously described by Hellwig et al. [17] and adapted with slight alterations.

In order to provide characterization evidence of the microvesicles released from the synovial sarcoma cells, microvesicles purified using the differential centrifugation steps described above were further analyzed by TEM. Briefly, after 5 min of adsorption, fixation of the microvesicle pellets was carried out for 5 min by the use of 1% glutaraldehyde (GA). The microvesicle pellets were then rinsed four times in ddH_2_O and negative staining was carried out for 1 min using 1% uranyl acetate. Negative staining is a method in which contrast is not applied to the object but to its environment, with the result resembling an inverted traditional TEM image [18]. Microvesicles were then digitally photographed as described above.

### 2.4. Nanoparticle Tracking Analysis (NTA)

NTA was carried out using the ZetaView® PMX 110 Nanoparticle Tracking Analyzer (Particle Metrix, Meerbusch, Germany) and the corresponding ZetaView® software. Camera shutter speed was maintained at 130 ms. Samples were diluted in sterile-filtered PBS to concentrations of 1 : 500 for purified synovial sarcoma microvesicles and 1 : 1000–1 : 2500 for purified serum microvesicles of patients with synovial sarcoma. Videos were recorded at 11 positions and 5 cycles with camera sensitivity ranging from 65% to 81%. Temperature was monitored manually and ranged from 21.0 to 22.0°C.

### 2.5. RNase A Treatment

To evaluate whether the fusion gene transcript was present inside the microvesicles, the pellet was dissolved in PBS and incubated for 30 min at 37°C with RNase A (Thermo Fisher Scientific, Waltham, MA, USA) at a final concentration of 100 *μ*g/mL or PBS as a negative control before RNA extraction as previously described by Skog et al.

### 2.6. Study Population

All patients included in this study received treatment from specialists in the interdisciplinary tumor board of the Comprehensive Cancer Center Freiburg (CCCF). Detection of the SYT-SSX fusion transcripts by chromogenic in situ hybridization (CISH), fluorescence in situ hybridization (FISH), or qPCR confirmed diagnosis of synovial sarcoma. Of the patients with active synovial sarcoma included in the study population, seven presented with metastasized disease, while one presented with localized disease of the lower extremity. Two of the patients received chemotherapy and one patient received radiotherapy within the last six weeks before blood withdrawal (Table 3). The control group consisted of healthy adults matched to the synovial sarcoma group in terms of age, sex, and body mass index (BMI) (Table 4).

### 2.7. Ethics, Consent, and Permissions

Signed informed consent was obtained from all participants, allowing analysis of blood samples, tumor tissue, and all clinical data. The Ethics Committee of the Albert-Ludwigs-University of Freiburg, Germany, approved the study. The design and performance of the study are in accordance with the Declaration of Helsinki.

### 2.8. Blood Sampling

All blood samples were collected by puncture of the antecubital vein without tourniquet through a 20-gauge needle. The first 3 mL of blood were discarded.

### 2.9. Whole Blood RNA

Each 2.5 mL of whole blood was collected and stabilized in PAXgene Blood RNA Tubes (PreAnalytiX, Hombrechtikon, Switzerland) as previously described by Keller et al. [19]. The RNA Tubes were incubated for at least 2 hours at room temperature (RT) after blood collection to ensure complete lysis of blood cells and were then stored at −20°C until further processing. Before starting the procedure, they were equilibrated to room temperature. Total RNA > 18 nucleotides (including miRNA) was purified manually using the PAXgene Blood miRNA Kit (PreAnalytiX) according to the manufacturer's protocol.

### 2.10. Separation of Mononuclear Cell Fraction

Approximately 9 mL of heparinized peripheral blood of patients with active synovial sarcoma was drawn and diluted with half the amount of PBS, then being gently overlaid on 4 mL Biocoll Separating Solution (Biochrom AG, Berlin, Germany) and centrifuged at 1200 g for 15 min at RT. The layer containing mononuclear cells was isolated from the interface, diluted with 10 mL PBS, and centrifuged at 300 g for 5 min. The supernatant was discarded and the pellet was dissolved in 4.5 mL FBS (fetal bovine serum superior; Biochrom AG), supplemented with 10% dimethyl sulfoxide (Sigma-Aldrich, St. Louis, MO, USA), then being stored at −80 degrees Celsius until further use.

### 2.11. Microvesicle-Purification of Serum Samples

7 mL of serum samples from healthy controls and synovial sarcoma patients was centrifuged at 2500 g for 15 min at RT. The supernatant was then further centrifuged at 2500 g for 15 min at RT. Microvesicles were subsequently pelleted by ultracentrifugation at 110,000 g for 80 min as described previously by Skog et al. [13] and washed once in PBS. The pellet was resolved in 15 *μ*L PBS before proceeding to RNA extraction.

### 2.12. RNA Isolation

Total RNA of both cells and microvesicles was purified using the RNeasy Mini Kit (Qiagen, Hilden, Germany) according to the manufacturer's protocol. RNA was quantified using the Nanodrop 2000 (Thermo Fisher Scientific).

### 2.13. Capillary Electrophoresis

RNA quality and quantity of cells and microvesicles were assessed by capillary electrophoresis using the Fragment Analyzer (Advanced Analytical Technologies GmbH, Heidelberg, Germany) and standard/high sensitivity RNA Analysis kits.

### 2.14. DNase-I Digestion and Conversion to cDNA

RNA was isolated and DNAse digestion and conversion to cDNA were carried out by the DNAse-I, amplification grade set (Life Technologies), and the AffinityScript Multi Temperature cDNA Synthesis Kit (Agilent Technologies, Santa Clara, CA, USA) according to the manufacturer's protocol. The reverse transcription reaction was incubated for 10 min at 25°C, followed by 1 h at 42°C and 15 min at 70°C.

### 2.15. qPCR

For detection of the SYT-SSX1 and SYT-SSX2 fusion gene, qPCR was performed using the Absolute qPCR ROX Mix (Thermo Fisher Scientific) and the following primers: SS18-SSX1 + FAM (Hs 03024820_ft), SS18-SSX2 + FAM (Hs03024398_ft) (TaqMan Gene Expression Assays; Life Technologies), and the GAPDH-Primer Set (GAPDH-probe 899, GAPDH-875F, and GAPDH 946-R; Eurofins MWG Operon, Huntsville, AL, USA) as the internal control. Briefly, the cycling conditions were enzyme activation at 95°C for 15 min, followed by 50 cycles of denaturation at 95°C for 15 s and annealing/extension at 60°C for 1 min. Ct-values < 39 were considered as positive.

### 2.16. Nested PCR

Nested PCR was carried out using the* Taq* PCR Core Kit (Qiagen) according to the manufacturer's protocol. The following PCR primers were used for the first-round PCR: 5-CAACAGCAAGATGCATACCA-3 and 5-CACTTGCTATGCACCTGATG-3. The primers of the second-round PCR were designed to amplify both SYT-SSX1 and SYT-SSX2 subtypes: 5-ACAGCCTGGACCACCACAGC-3 and 5-AGGCATGTTTCCCCCTTTTG-3, yielding PCR products of 212 base pairs (bp). The primer sequences were adapted from Hashimoto et al. [20]. Cycling conditions were 35 cycles of denaturation at 94°C for 40 s, annealing at 50°C for 1 min, and extension at 72°C for 1 min after an initial denaturation step of 94°C for 3 min. Afterwards, a final extension step was carried out at 72°C for 10 min.

### 2.17. Nested qPCR

qPCR was performed of the second-round PCR product of the nested PCR from whole blood, microvesicle, and the mononuclear cell fraction of synovial sarcoma and healthy donors using the SS18-SSX1 + FAM (Hs 03024820_ft) and SS18-SSX2 + FAM (Hs03024398_ft) primers.

### 2.18. Droplet Digital PCR (ddPCR)

Droplet digital PCR was carried out using the SS18-SSX1 + FAM (Hs 03024820_ft) and SS18-SSX2 + FAM (Hs03024398_ft) primers and the QX100 ddPCR system (Bio-Rad, Hercules, CA, USA) according to the manufacturer's protocol. Hereby, PCR amplification is carried out within each droplet using a thermal cycler after partitioning of samples into droplets by the QX100 droplet generator. After PCR, droplets are streamed in a single file on a QX100 droplet reader, which counts the fluorescent positive and negative droplets to calculate target RNA concentration. Event counts < 5 were interpreted as not detected, since negative controls showed up to five events.

### 2.19. Statistics

*p* values below 0.05 were considered statistically significant. Statistical analysis was carried out using Student's *t*-test for independent samples.

Data were presented as mean value ± standard error of mean (SEM). All data were analyzed with GraphPad Prism 6.0 (GraphPad Software, San Diego, CA, USA).

## 3. Results and Discussion

**Figure 1:**
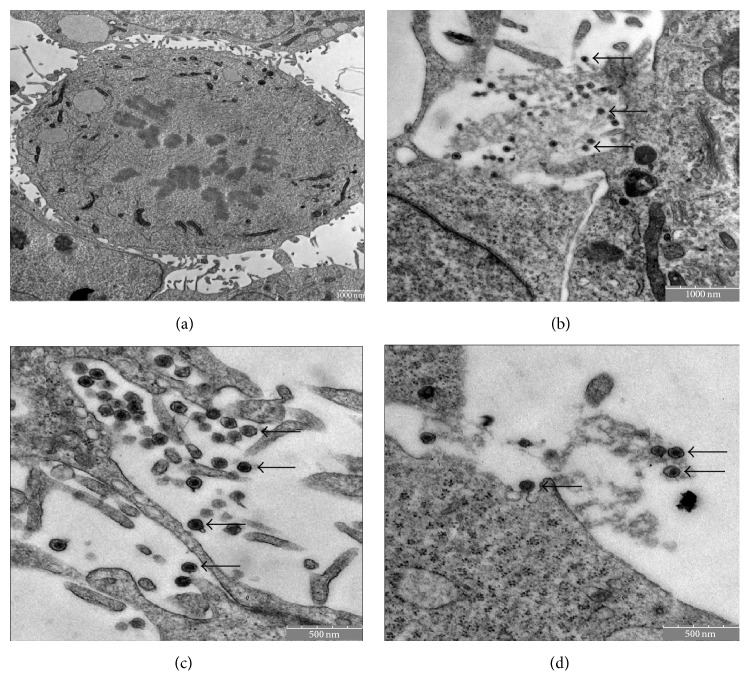
Visualization of synovial sarcoma cells and microvesicles by transmission electron microscopy (TEM). (a) Synovial sarcoma cell. Bar indicating 1000 nm. ((b)–(d)) Close-up view which shows the release of microvesicles (arrows) by the synovial sarcoma cell. Bars indicating 1000 nm (b) and 500 nm ((c) and (d)).

**Figure 2:**
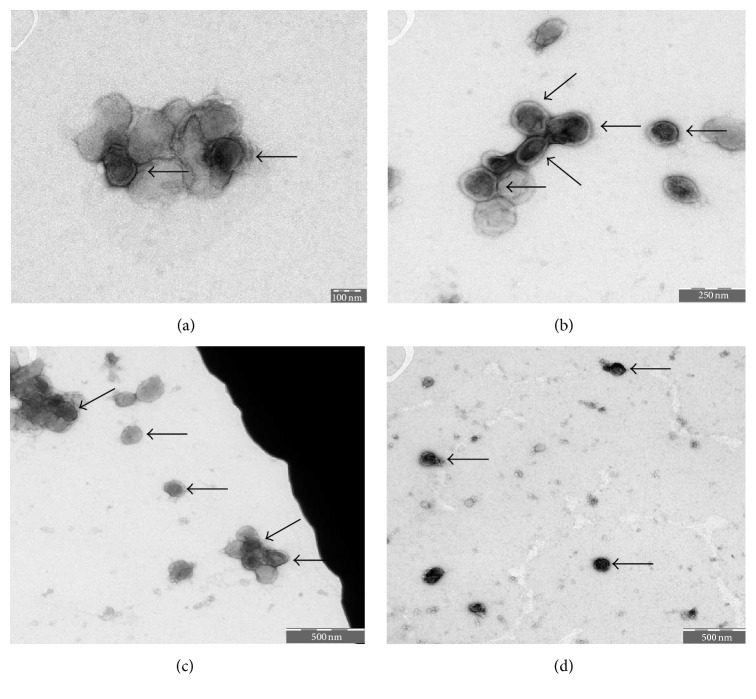
Visualization of purified synovial sarcoma microvesicles (arrows) by transmission electron microscopy (TEM). (a) Bar indicating 100 nm. (b) Bar indicating 250 nm. ((c) and (d)) Bar indicating 500 nm.

Electron microscopy showed synovial sarcoma cells releasing microvesicles enclosed by a protective membrane (Figures 1(a)–1(d)). Synovial sarcoma microvesicles purified by differential centrifugation steps were further analyzed by electron microscopy (Figure 2) and were shown to correspond to the microvesicles released from synovial sarcoma cells as depicted in Figures 1(a)–1(d) in size and aspect.

**Figure 3:**
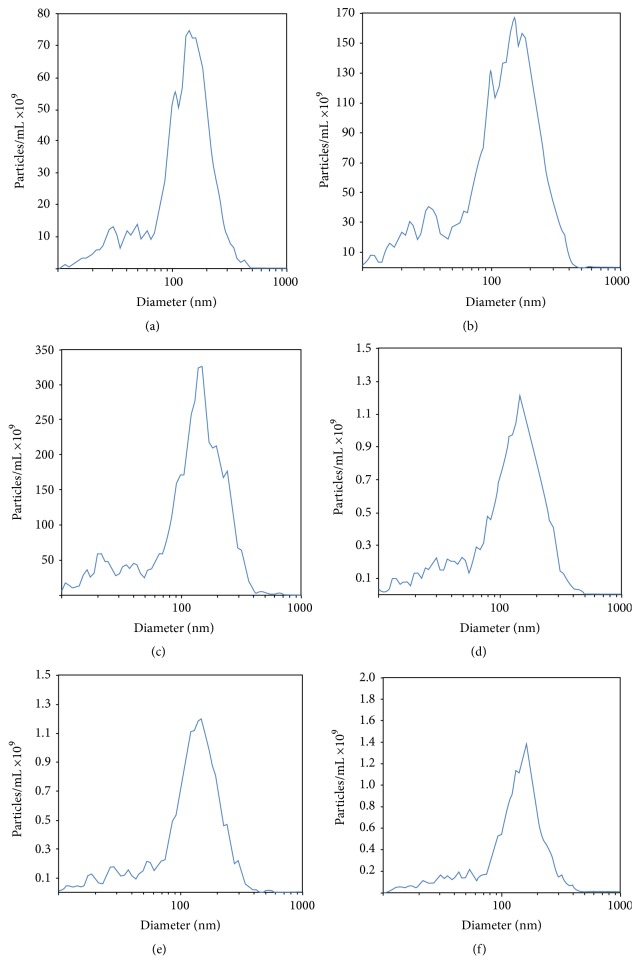
Nanoparticle Tracking Analysis (NTA) of microvesicles purified from serum of patients with active synovial sarcoma ((a)–(c)) and synovial sarcoma cells ((d)–(f)). Mean diameter peaks were 151.7 nm for microvesicles extracted from synovial sarcoma patient serum ((a)–(c)) and 154.4 nm ((d)–(f)) for microvesicles purified from synovial sarcoma cells. Mean concentration levels were 3155.0 × 10^9^ particles/mL in serum of patients with active synovial sarcoma ((a)–(c)) and 18.60 × 10^9^ particles/mL in synovial sarcoma cell supernatant ((d)–(f)).

To further characterize the microvesicles, Nanoparticle Tracking Analysis (NTA) was carried out. Microvesicles purified from serum of patients with active synovial sarcoma showed similar mean diameter peaks when compared to microvesicles purified from synovial sarcoma cells (151.7 nm (Figures 3(a)–3(c)) and 154.4 nm (Figures 3(d)–3(f)), resp.). Mean concentration levels of microvesicles in serum of patients with active synovial sarcoma and synovial sarcoma cell supernatant were 3155.0 × 10^9^ particles/mL (Figures 3(a)–3(c)) and 18.60 × 10^9^ particles/mL (Figures 3(d)–3(f)), respectively.

**Figure 4:**
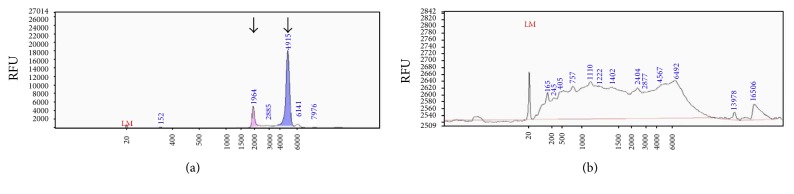
Differences in RNA size distribution (a) RNA of 1273/99 synovial sarcoma cells showing the characteristic ribosomal peaks of cellular RNA. The two prominent peaks (arrows) represent 18S (left) and 28S (right) ribosomal RNA. (b) RNA of microvesicles lack the characteristic ribosomal 18S and 28S peaks.

Performing bioanalysis of RNA from synovial sarcoma microvesicles and their cells of origin, significant differences in RNA size distribution were found, with microvesicle RNA lacking the characteristic ribosomal RNA peaks of cellular RNA (Figure 4).

**Figure 5:**
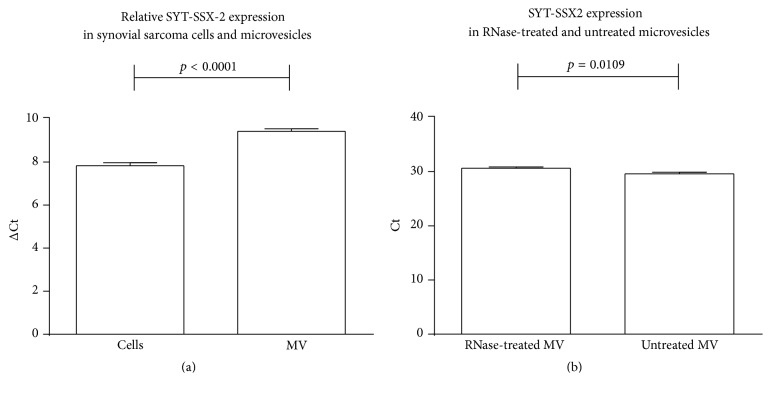
(a) Relative expression of the SYT-SSX2 fusion gene transcript in synovial sarcoma cells and microvesicles, normalized to GAPDH. (b) Expression of the SYT-SSX2 fusion gene transcript in microvesicles treated with RNase A and untreated microvesicles. MV: microvesicles.

In vitro, the SYT-SSX2 fusion gene transcript was detected in both synovial sarcoma cells and microvesicles (*n* = 3) (Figure 5(a)), with microvesicle RNase A treatment showing only a small decrease of the fusion gene mRNA compared to untreated microvesicles (*n* = 3) (Figure 5(b)), thus showing that the mRNA is contained inside the microvesicles, being protected from the RNase by the lipid bilayer.

When comparing the sensitivity of nested qPCR, qPCR, nested PCR, and droplet digital PCR for detection of the SYT-SSX2 fusion gene transcript in synovial sarcoma cells and microvesicles, nested qPCR and qPCR showed the highest sensitivity for the detection of the fusion gene transcript in both microvesicles and cells, whereas ddPCR showed the lowest sensitivity (Tables 1 and 2).

We then employed different assays for detection of the SYT-SSX fusion transcripts to peripheral blood samples of patients with synovial sarcomas. Analysis of corresponding tumor tissue revealed that two patients presented the SYT-SSX2 fusion gene phenotype, while five presented the SYT-SSX1 phenotype [21], which has been described as more common [10, 22]. Tumor tissue of one patient was not available for analysis. Information regarding disease and therapy status of sarcoma patients is illustrated in Table 3. Synovial sarcoma patients (*n* = 8) did not differ significantly from healthy controls (*n* = 5) concerning age, BMI, hemoglobin (Hb) level, platelet count, and leukocyte count (Table 4).

**Figure 6:**
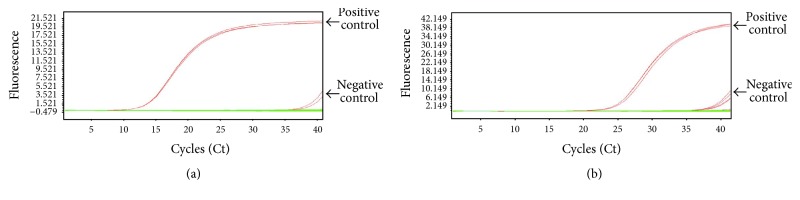
Analysis of the presence of the SYT-SSX fusion gene in whole blood, the mononuclear cell fraction, and serum microvesicles of synovial sarcoma patients by nested qPCR (a) and qPCR (b). Synovial sarcoma cells: positive control. Negative controls showed positivity at Ct-cycles ≥ 39. Patient samples showed Ct-cycles > 39 and were thus interpreted as negative.

**Figure 7:**
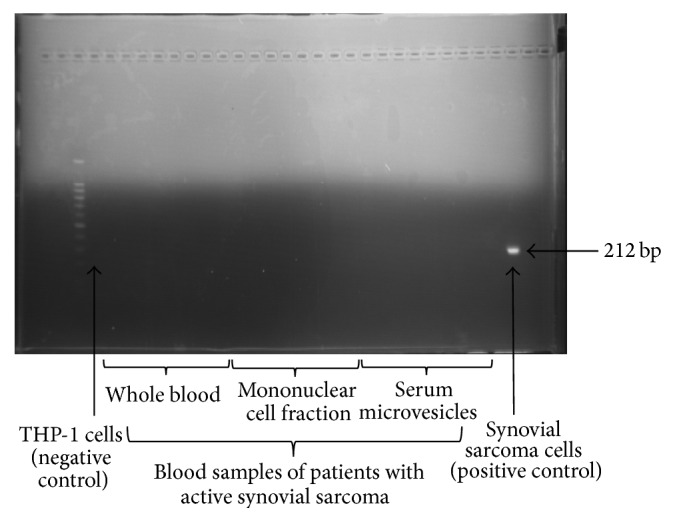
Analysis of the presence of the SYT-SSX fusion gene in whole blood, the mononuclear cell fraction, and serum microvesicles of synovial sarcoma patients by nested PCR. THP-1 cells: negative control. 1273/99 synovial sarcoma cells: positive control, showing the SYT-SSX fusion gene transcript (212 bp; the primers were designed to amplify both SYT-SSX1 and SYT-SSX2 subtypes [20]).

**Figure 8:**
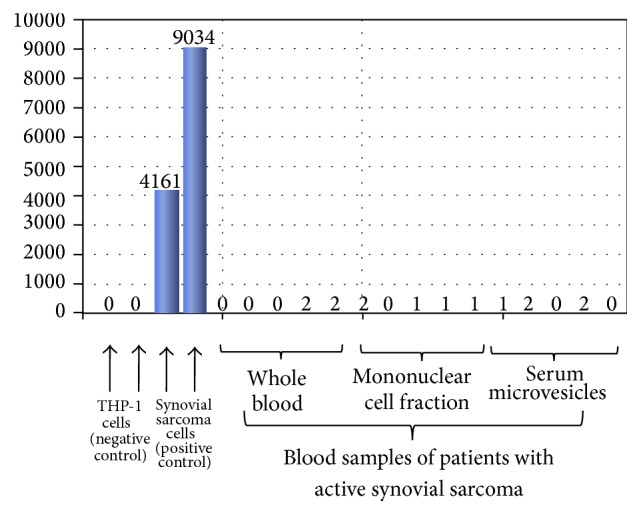
Analysis of the presence of the SYT-SSX fusion gene in whole blood, the mononuclear cell fraction, and serum microvesicles of synovial sarcoma patients by ddPCR.

Nested qPCR (Figure 6(a)), qPCR (Figure 6(b)), nested PCR (Figure 7), and ddPCR (Figure 8) did not detect the SYT-SSX1/2 fusion gene transcripts in the extracted whole blood, mononuclear cells, and microvesicles of synovial sarcoma patients and healthy donors.

Thus, we could show that synovial sarcoma cells release small vesicles harboring the synovial sarcoma cell-specific fusion gene transcript SYT-SSX. Hereby, the size distribution of RNA contained in these microvesicles differs significantly to their cells of origin. As shown by its resistance to RNase treatment, the fusion gene transcript seems to be located inside the protective microvesicle membrane. To our knowledge, this is the first study to show that the SYT-SSX fusion transcript is present not only in synovial sarcoma cells, but also in synovial sarcoma microvesicles.

Since it has been shown that tumor microvesicles act as intercellular messengers, activate signaling pathways, and modulate cell survival when being engulfed by other cells such as monocytes [23], synovial sarcoma microvesicles might serve as important tumor mediators, which interact with immune cells in the tumor's periphery and throughout the circulation as well as with nearby host tissues. In this context, Andreola et al. showed that tumor cells release Fas ligand-bearing microvesicles, which trigger Fas-dependent apoptosis of lymphoid cells, thus impairing the efficacy of antitumor immune responses, a mechanism known as “Fas tumor counterattack” [24]. This finding suggests that one of the major roles of the detected tumor microvesicles might be the role of “guardsmen” interfering with lymphocytes and other immune cells and impeding them from applying their antitumor activity. As it could be proven that tumor cells with highly metastatic potential release a greater amount of tumor microvesicles than cells with low metastatic ability [25], microvesicles seem the ideal biomarkers for the detection of tumor activity. This could further be underlined by the fact that the detection of the synovial sarcoma-specific fusion gene in microvesicles can be detected in much lower total RNA concentrations than in their cells of origin (Tables 1 and 2). Nested qPCR was shown to be by far the most sensitive method for detecting the SYT-SSX fusion gene transcript, followed by qPCR, nested PCR, and ddPCR (Tables 1 and 2). This is in line with a study conducted by Amary et al., which revealed that, when employing qPCR primers in a conventional PCR assay, the SYT-SSX fusion gene can be found in approximately 50% of cases initially classified as negative for SYT-SSX, furthermore showing that qPCR shows the highest sensitivity when compared to conventional RT-PCR and FISH. However, this might be based on the PCR primer design and probably also the size of the product rather than the method of detection [26]. By combining nested PCR and qPCR, we could further increase the sensitivity of the SYT-SSX fusion gene detection by several magnitudes, which might be useful for diagnosis of synovial sarcoma, when only very small tumor samples are available, for example, after core needle biopsy.

Still, qPCR did not prove to always be superior to ddPCR in other applications. Drandi et al. found ddPCR comparable with qPCR when detecting immunoglobulin gene rearrangement and BCL2/immunoglobulin gene major breakpoint region rearrangement in multiple myeloma, mantle cell lymphoma, and follicular lymphoma [27]. Fontanelli et al. showed slightly higher sensitivity of ddPCR for the detection of the JAK2V617F mutation in Philadelphia-negative chronic myeloproliferative neoplasms compared to qPCR [28]. However, when assessing cytomegalovirus load in clinical samples, qPCR showed greater sensitivity than did ddPCR [29]. Also, in accordance with our study, Kiselinova et al. showed that a major disadvantage of ddPCR is the high number of false-positive results when comparing ddPCR and seminested qPCR for quantification of unspliced and multiply spliced HIV-1 RNA, as no-template controls were consistently negative in the seminested qPCR but yielded positive ddPCR signals [30]. Thus, it seems that the sensitivity of each method varies throughout different studies, probably being primarily dependent on the PCR primer design and size of the product.

To date, there are only few studies examining potential biomarkers of sarcoma. Recently, specific miRNA profiles were found in the peripheral blood of patients with synovial sarcoma [21] and rhabdomyosarcoma [31]. Also, research has been sparked into the field of circulating tumor cells, which have been detected in several malignancies [32, 33] and which were shown to correlate with stage of disease and presence of metastases [34] as well as with progression-free and overall survival [35] of different neoplasms. Satelli et al. have examined cell-surface vimentin as a universal marker on circulating sarcoma cells using a monoclonal antibody, confirming the positivity of circulating tumor cells of sarcoma patients through blood spiking assays and immunofluorescence staining [36]. However, vimentin has been described in various other malignancies [37, 38], thus hardly serving as a specific biomarker.

As described in a case-report, Hashimoto et al. managed to detect tumor cells expressing the SYT-SSX fusion gene transcript in the peripheral blood of a 22-year-old pregnant woman with synovial sarcoma by nested PCR [20]. Thus, a more specific way of analyzing the presence of circulating tumor cells in sarcomas could be the detection of specific fusion gene transcripts such as the SYT-SSX fusion gene mRNA in synovial sarcoma, the EWS-ERG and EWS-FLI1 fusion transcript in Ewing sarcoma (EWS) and Primitive Neuroectodermal Tumor (PNET) [39–41], the PAX3-FKHR or PAX7-FKHR fusion transcript in alveolar rhabdomyosarcoma [42], or the ASPSCR1–TFE3 fusion transcript in alveolar soft part sarcoma (ASPS) [43]. Although Hashimoto et al. detected the SYT-SSX fusion gene product in the peripheral blood of a pregnant woman with a large synovial sarcoma of the thigh before resection of the sarcoma, the SYT-SSX fusion gene transcript could not be detected after the development of lung metastases, showing that the circulating tumor cells were reduced to an undetectable level after tumor resection [20].

Circulating tumor cells carrying a sarcoma-specific mRNA fusion gene transcript were detected only in few sarcoma patients. Schleiermacher et al. detected circulating tumor cells in the peripheral blood of 22% of patients with metastatic and 20% of patients with localized Ewing sarcoma [39]. Hoshino et al. detected the specific fusion gene transcript of ASPS, ASPSCR1-TFE3, in the peripheral blood sample of 1 ASPS patient with distant metastases [43], while Kelly et al. did not detect the PAX3-FKHR or PAX7-FKHR fusion transcript specific for alveolar rhabdomyosarcoma in any of the peripheral blood samples of alveolar rhabdomyosarcoma patients but in bone marrows of a minority of the patients [42]. This shows that, in many sarcoma patients, circulating tumor cells are reduced to an undetectable level.

The fact that Skog et al. detected the tumor-specific EGFRvIII mRNA variant specific for glioblastoma in serum microvesicles of glioblastoma patients [13] further supports our finding that circulating tumor-derived microvesicles carry tumor-specific mRNA, thus potentially serving as highly specific tumor biomarkers. Skog et al. furthermore showed that tumor-derived microvesicles deliver genetic information and proteins to surrounding cells in the tumor periphery and induce proliferation of human glioma cells.

Thus, as tumor-specific mutant mRNA can be detected in serum microvesicles from glioblastoma patients, tumor-derived microvesicles may be a helpful tool in diagnosis as well as in therapeutic decisions for patients with malignant diseases.

This is especially important as these tumor-specific mRNAs can enable highly sensitive detection of tumor microvesicles, as we were able to detect the SYT-SSX fusion transcript by nested qPCR in total microvesicle RNA of only 6 pg (Table 2), which is less than the RNA content of a single cell.

Although we could prove that the SYT-SSX fusion gene transcript was present in synovial sarcoma microvesicles, the fusion transcript could not be detected in microvesicles isolated from the peripheral blood of synovial sarcoma patients. Thus, although tumor microvesicles seem to be ideal biomarkers for synovial sarcoma, more sensitive methods need to be developed for their detection in the peripheral circulation.

## 4. Conclusions

Synovial sarcoma cells release microvesicles which harbor the SYT-SSX fusion transcript inside their protective membrane. These vesicles might serve as a diagnostic biomarker; however, more sensitive assays are needed to detect cancer-specific microvesicles in the peripheral blood of cancer patients.

## Figures and Tables

**Table 1:** Comparison of sensitivity of nested PCR, qPCR, nested PCR, and ddPCR in the detection of the SYT-SSX2 fusion gene in synovial sarcoma cells. D: detected, ND: not detected.

Amount of total RNA in ng (synovial sarcoma cells)	Nested qPCR	qPCR	Nested PCR	ddPCR
25,000	D	D	D	D
12,500	D	D	D	D
6,250	D	D	D	ND
3,125	D	D	D	ND
1,562	D	D	ND	ND
0,781	D	D	ND	ND
0,391	D	ND	ND	ND
0,195	D	ND	ND	ND
0,098	ND	ND	ND	ND
0,049	ND	ND	ND	ND
0,024	ND	ND	ND	ND
0,012	ND	ND	ND	ND
0,006	ND	ND	ND	ND
0,003	ND	ND	ND	ND

**Table 2:** Comparison of sensitivity of nested PCR, qPCR, nested PCR, and ddPCR at detection of SYT-SSX fusion gene in 1273/99 synovial sarcoma microvesicles. D: detected, ND: not detected.

Amount of total RNA in ng (synovial sarcoma microvesicles)	Nested qPCR	qPCR	Nested PCR	ddPCR
25,000	D	D	D	D
12,500	D	D	D	D
6,250	D	D	D	D
3,125	D	D	D	ND
1,562	D	D	ND	ND
0,781	D	D	ND	ND
0,391	D	ND	ND	ND
0,195	D	ND	ND	ND
0,098	D	ND	ND	ND
0,049	D	ND	ND	ND
0,024	D	ND	ND	ND
0,012	D	ND	ND	ND
0,006	D	ND	ND	ND
0,003	ND	ND	ND	ND

**Table 3:** Disease and therapy status of patients with synovial sarcoma. M_0_: local disease, M_1_: metastatic disease. Current chemotherapy/radiotherapy/anticoagulation involves treatment within the last 6 weeks.

Synovial sarcoma patients	M_0_	M_1_	Current chemotherapy	Current radiotherapy	Anticoagulation	Fusion gene type
Patient 1	X			X		SS18-SSX1
Patient 2		X	X			SS18-SSX2
Patient 3		X	X		X	No tumor tissue available
Patient 4		X				SS18-SSX1
Patient 5		X				SS18-SSX1
Patient 6		X			X	SS18-SSX1
Patient 7		X				SS18-SSX2
Patient 8		X				SS18-SSX1

**Table 4:** Demographic patient data (age, body mass index (BMI), and blood count (Hb: hemoglobin level, platelet count, and leukocyte count)) of synovial sarcoma patients. Data are presented as mean value ± standard error of mean (SEM).

Patients	Age (years)	BMI (kg/m^2^)	Hb (g/dL)	Platelets (×10^6^/L)	Leukocytes (×10^6^/L)
Patients with synovial sarcoma (*n* = 8)	50.00 ± 4.255	23.08 ± 1.146 *N* = 8	13.23 ± 0.6187	262.6 ± 61.43	7.245 ± 1.382
Healthy controls (*n* = 5)	51.20 ± 4.893	23.62 ± 1.301	14.98 ± 0.5490	174.0 ± 15.53	5.468 ± 0.4835
*p* value	0.8599	0.7682	0.0773	0.2911	0.3488

## Abbreviations

ASPS: Alveolar soft part sarcoma
BMI: Body mass index
CISH: Chromogenic in situ hybridization
dFBS: Microvesicle-depleted fetal bovine serum
EWS: Ewing sarcoma
FISH: Fluorescence in situ hybridization
ddPCR: Droplet Digital Polymerase Chain Reaction
Hb: Hemoglobin
NTA: Nanoparticle Tracking Analysis
PCR: Polymerase Chain Reaction
PNET: Primitive Neuroectodermal Tumor
qPCR: Quantitative Polymerase Chain Reaction (real-time)
RT: Room temperature
RT-PCR: Reverse Transcription-Polymerase Chain Reaction
TEM: Transmission electron microscopy.
